# Anatomical basis of a safe mini-invasive technique for lengthening of the anterior gastrocnemius aponeurosis

**DOI:** 10.1007/s00276-020-02536-1

**Published:** 2020-07-23

**Authors:** Simone Moroni, Alejandro Fernández-Gibello, Gabriel Camunas Nieves, Ruben Montes, Marit Zwierzina, Teresa Vazquez, Maria Garcia-Escudero, Fabrice Duparc, Bernhard Moriggl, Marko Konschake

**Affiliations:** 1 Faculty of Health Sciences At Manresa, Department of Podiatry, Universidad de Vic—Universidad Central de Catalunya (UVic-Ucc), Clinic Vitruvio Biomecánica, BarcelonaMadrid, Spain; 2 Faculty of Health Sciences, Department of Podiatry, University of La Salle, Clinic Vitruvio Biomecánica, Madrid, Spain; 3 Universidad La Salle, Centro adscrito a la Universidad Autónoma de Madrid, Madrid, Spain; 4 Vitruvio Biomecanica Y Cirugia Clinic, Madrid, Spain; 5 Department of Plastic, Reconstructive and Aesthetic Surgery, Center of Operative Medicine, Medical University of Innsbruck (MUI), Innsbruck, Austria; 6 Anatomy and Embryology Department, School of Medicine, Complutense University of Madrid, Madrid, Spain; 7 School of Physiotherapy and Podiatry, University Catolica de Valencia, Valencia, Spain; 8 Laboratory of Anatomy, Faculty of Medicine, Rouen-Normandy University, Rouen, France; 9 Department of Anatomy, Histology and Embryology, Institute of Clinical and Functional Anatomy, Medical University of Innsbruck (MUI), Müllerstr. 59, 6020 Innsbruck, Austria

**Keywords:** Ultrasound, Minimally invasive, Gastrocnemius muscle, Aponeurosis

## Abstract

**Background:**

The surgical procedure itself of lengthening the gastrocnemius muscle aponeurosis is performed to treat multiple musculoskeletal, neurological and metabolical pathologies related to a gastro-soleus unit contracture such as plantar fasciitis, Achilles tendinopathy, metatarsalgia, cerebral palsy, or diabetic foot ulcerations. Therefore, the aim of our research was to prove the effectiveness and safety of a new ultrasound-guided surgery-technique for the lengthening of the anterior gastrocnemius muscle aponeurosis, the “GIAR”- technique: the gastrocnemius-intramuscular aponeurosis release.

**Methods and results:**

An ultrasound-guided surgical GIAR on ten fresh-frozen specimens (10 donors, 8 male, 2 females, 5 left and 5 right) was performed. Exclusion criteria of the donated bodies to science were BMI above 35 (impaired ultrasound echogenicity), signs of traumas in the ankle and crural region, a history of ankle or foot ischemic vascular disorder, surgery or space-occupying mass lesions. The surgical procedures were performed by two podiatric surgeons with more than 6 years of experience in ultrasound-guided procedures. The anterior gastrocnemius muscle aponeurosis was entirely transected in 10 over 10 specimens, with a mean portal length of 2 mm (± 1 mm). The mean gain at the ankle joint ROM after the GIAR was 7.9° (± 1.1°). No damages of important anatomical structures could be found.

**Conclusion:**

Results of this study indicate that our novel ultrasound-guided surgery for the lengthening of the anterior gastrocnemius muscle aponeurosis (GIAR) might be an effective and safe procedure.

## Introduction

The term "gastrocnemius-soleus complex", the “triceps surae” as officially named in the International Anatomical Terminology, has been used in daily routine clinical practice for many decades, consisting from the gastrocnemius, the soleus and the plantaris muscle of the crus; the literature shows that this complex is present regularly in the general population, while the small plantaris muscle itself is variable and, according to literature, can be absent in up to 50% of the patients [10, 11]. The prevalence of a superficial posterior compartment (SPC)-contracture in patients with symptomatic musculoskeletal pathologies of the mid- and the forefoot has been evaluated with around 88–96.5% versus 44% in control groups [9, 12]. Hill et al. described that in 10% of all patients suffering from diabetes an equinus deformity is present [12]: the equinus deformity has correlations with changes in plantar pressures exerted during weightbearing activities leading to a great risk factor for the development of forefoot plantar foot ulcers [9].

The gastrocnemius muscle lengthening was firstly described in 1913 by Vulpius and Stoeffel [2], followed by other authors such as Silverskiold, Baumann, Strayer, Baker, Hoke, White or Paley, who described the surgical approaches at different anatomical gastrocnemius muscle levels [3, 6, 18, 27, 33].

The surgical procedure itself of lengthening the gastrocnemius muscle aponeurosis is performed to treat multiple musculoskeletal, neurological and metabolical pathologies related to a SPC contracture such as plantar fasciitis, Achilles tendinopathy, metatarsalgia, cerebral palsy, or diabetic foot ulcerations [7, 15].

Recently, different techniques of minimally invasive gastrocnemius muscle recessions have been described [4, 19, 30], only one performed via ultrasound-guidance, which consists in transecting the gastrocnemius tendon through the so called “Strayer technique” at the anatomical gastrocnemius muscle lengthening level III [31].

In contrast to the “Strayer approach”, the “Baumann technique” targets the anterior gastrocnemius muscle aponeurosis, consisting of an intramuscular aponeurosis transection, also re-named by Blitz et al. by the acronym “GIAR”: Gastrocnemius -intramuscular-aponeurosis-recession [7, 8]. The Baumann procedure is performed at the gastrocnemius muscle level IV and leads to lower rates of complications and less weakness [12]. Furthermore, it could be shown that the “Strayer technique” has less stability in lengthening with respect to the Baumann and the Barouk procedures, but has similar outcomes in terms of ankle range of motion (ROM) correction [15, 25].

To the best of our knowledge, until now, a new ultra-minimally invasive, ultrasound-guided surgical approach for the lengthening of the anterior gastrocnemius muscle aponeurosis has not yet been described. Therefore, the primary aim of our research was to prove the effectiveness and safety of a new ultrasound-guided surgery-technique for the lengthening of the anterior gastrocnemius muscle aponeurosis, the “GIAR”- technique: the gastrocnemius-intramuscular aponeurosis release. Our secondary aim was to ascertain the gain in ROM at the ankle joint after the procedure.

## Material and methods (Figs. 1, 2, 3, 4, 5)

**Fig. 1:**
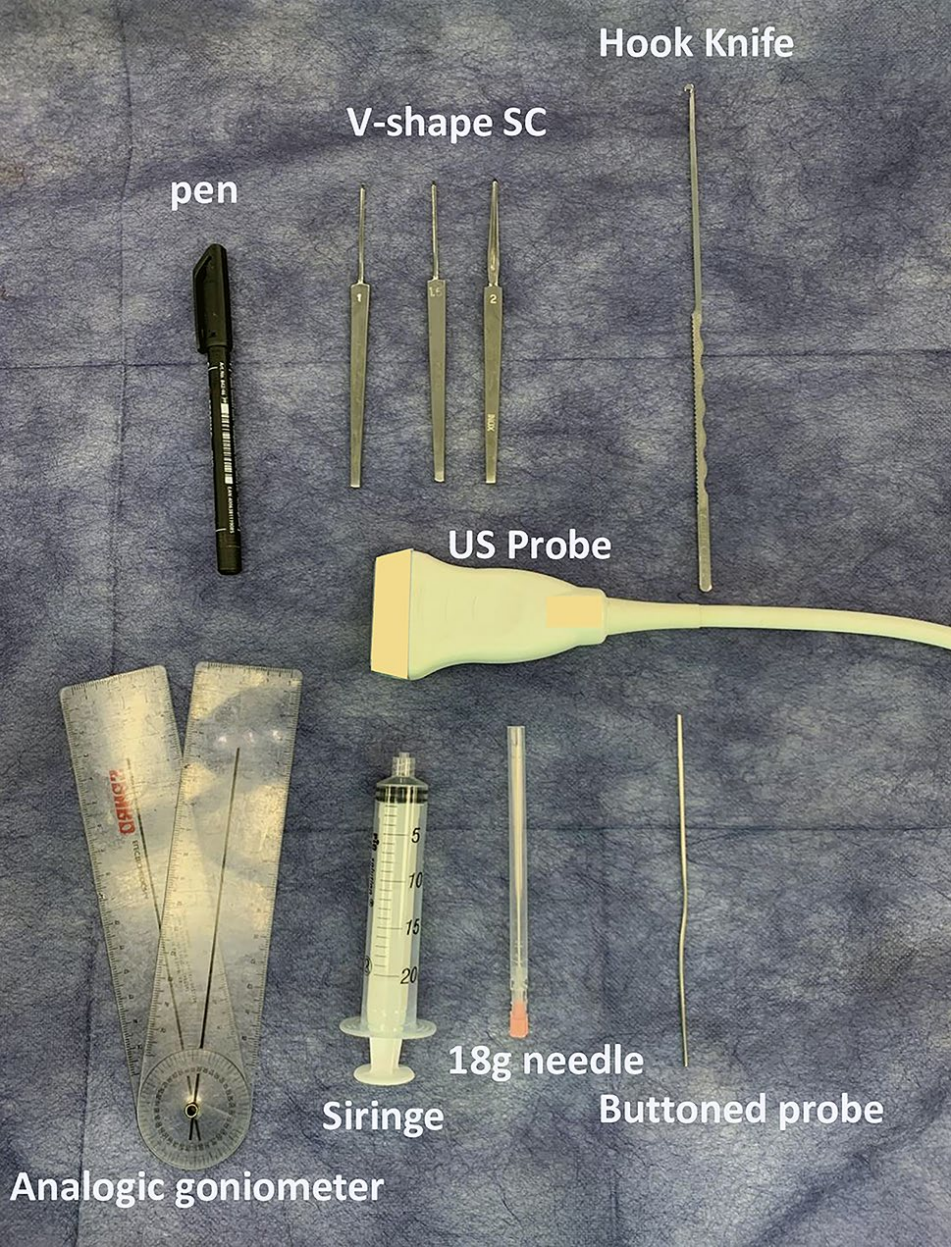
Instruments for the minimally invasive ultrasound-guided procedure. High-resolution ultrasound; 18-gauge needle; 50 cc syringe; V-shape straight curette; hook knife (Acufex®), analogic goniometer, surgical pen

**Fig. 2:**
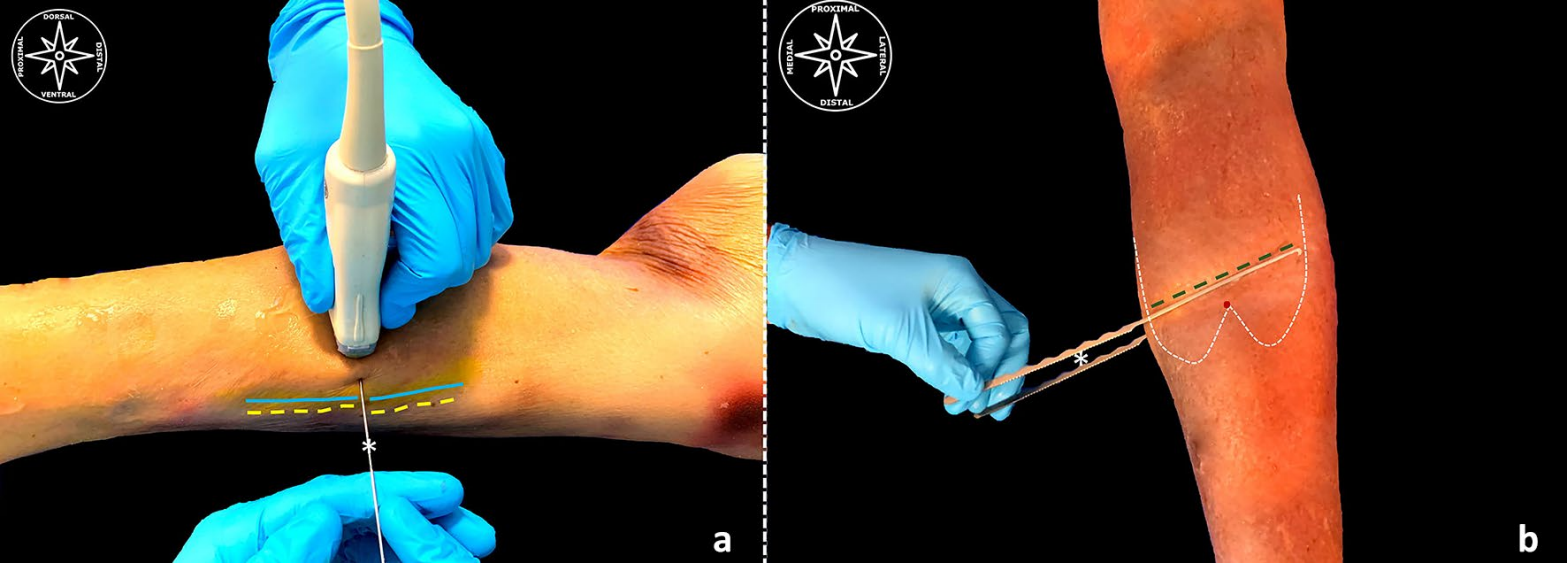
**a**, **b** US-guided pre-surgical mapping for ultrasound-guided GIAR. **a** (yellow line)… mapping of the course of saphenous nerve along the crural region; (Blue line)…mapping of the course of great saphenous vein along the crural region; (white asterisk)…the needle is inserted through the surgical portal immediately dorsal to the course of the great saphenous vein, superficial at the interval between the anterior gastrocnemius aponeurosis and the soleus muscle. **b** (white dotted lines)…gastrocnemius muscle bellies representation at the dorsal aspect of the crural region. (white asterisk)…the hook knifes, ventral one introduced through the surgical portal at the interval between the soleus and gastrocnemius muscle aponeurosis (up to the most lateral aspect), the other one positioned at the dorsal aspect of the skin of the crural region as a demonstration for the correct positioning of the first one. (Red dot)… represents the distal transection point. (green dotted line)…represents the transection at the anterior gastrocnemius muscle aponeurosis (color figure online)

**Fig. 3:**
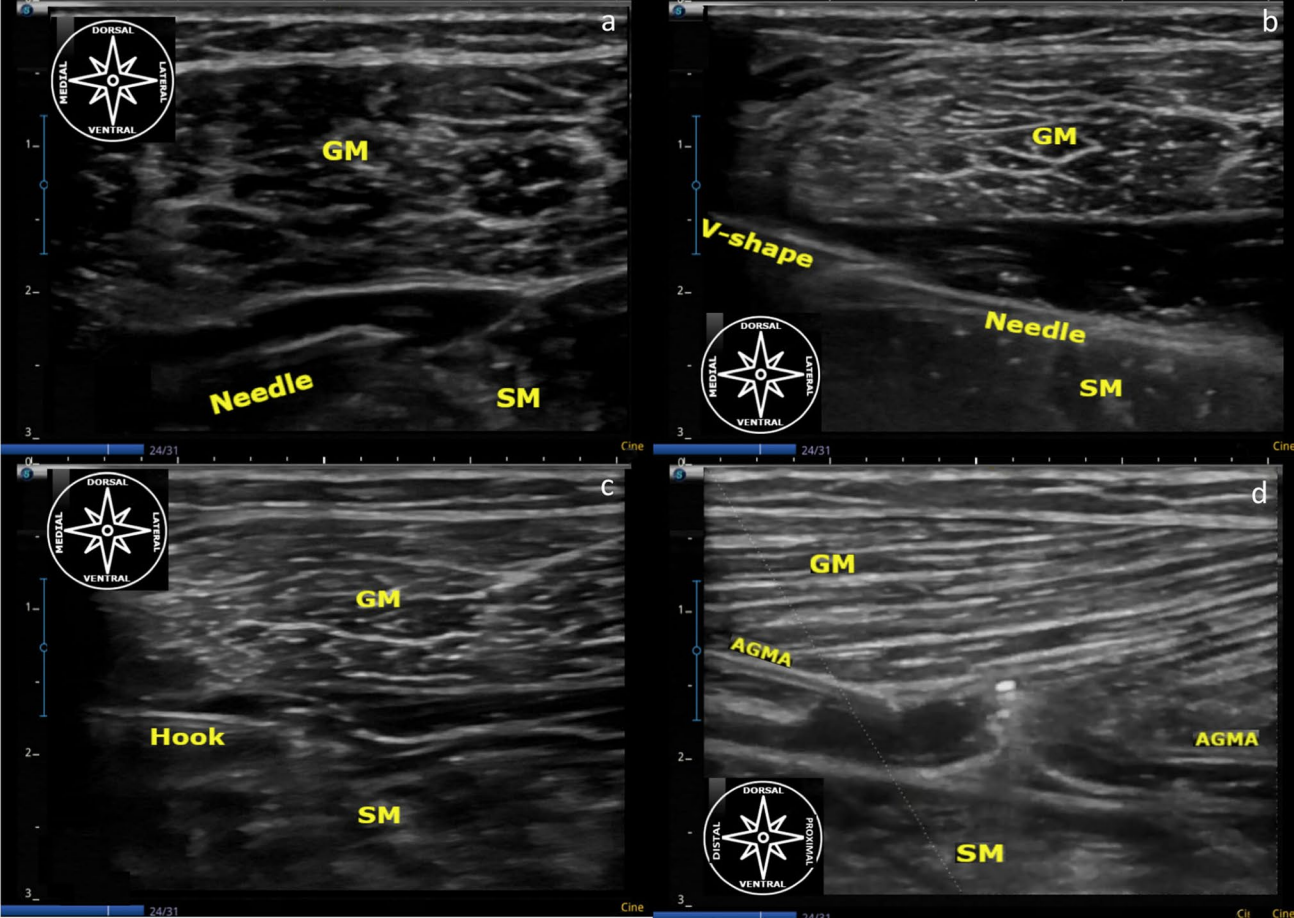
**a**–**d** US-guided surgical routine for GIAR. 3a (needle)…18 g needle seen through an in-plane approach in the long axis hydrodissecting proximal to the conjoint tendon, the virtual anatomical space in between the anterior gastrocnemius muscle aponeurosis, ventrally to both gastrocnemius heads from medial to lateral and the soleus aponeurosis and its underlying muscle belly (SM) dorsally, both seen in the short axis. **b** from the medial portal, it could be seen the V shape straight courette (V-shape) dorsal to the 18 g needle (needle) both seen through an in-plane approach in the long axis at the hydrodissected interval in between the anterior gastrocnemius muscle aponeurosis and the medial head of its muscle belly (GM) and soleus aponeurosis and its underlying muscle belly (SM) both seen in the short axis. **c** from the medial portal, it can be seen the hook knife through an in plane approach in the long axis at the hydrodissected interval in between the anterior gastrocnemius muscle aponeurosis and medial head of its muscle belly (GM) and the soleus aponeurosis and its underlying muscle belly (SM) both seen in the short axis. **d** After hook knife transection it can be seen the gap at the anterior gastrocnemius muscle aponeurosis (AGMA) and the buttoned probe in between the gap through an out of plane approach in the short axis at the hydrodissected interval in between the anterior gastrocnemius muscle aponeurosis and medial head of its muscle belly (GM) and soleus aponeurosis and its underlying muscle belly (SM) both seen in the long axis (color figure online)

**Fig. 4:**
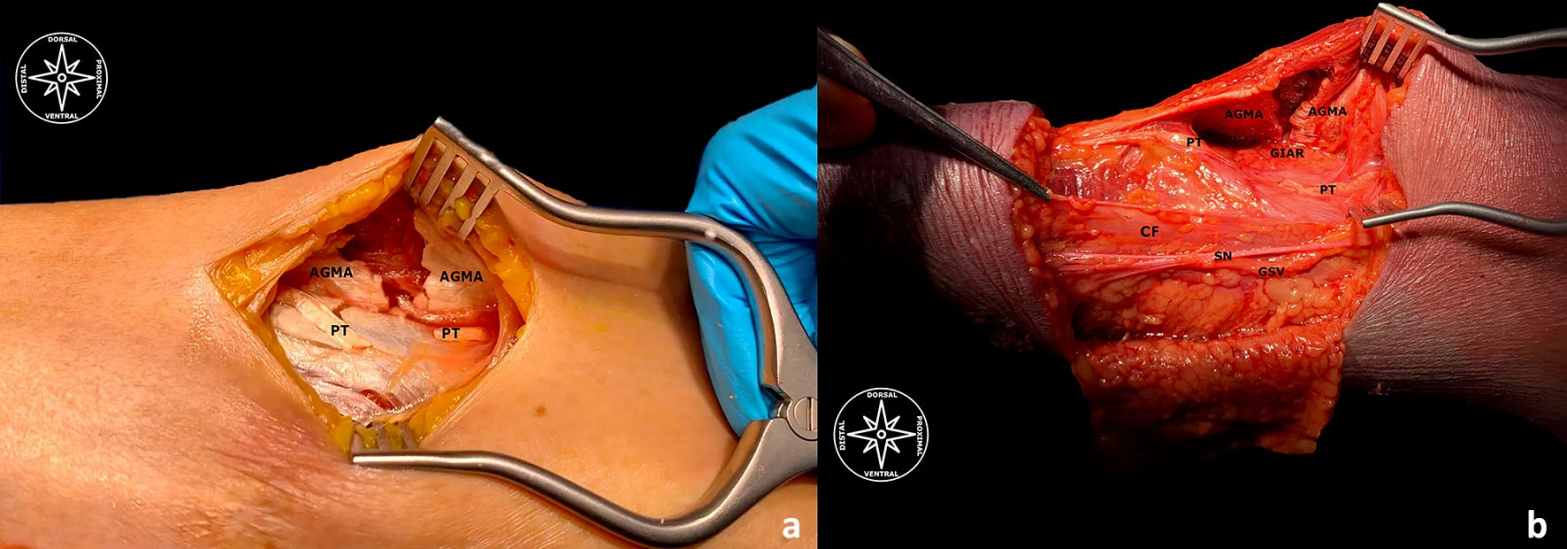
**a**, **b** Anatomical dissections for proof after US-guided GIAR technique. **a** (AGMA)… transected anterior gastrocnemius muscle aponeurosis; (PT)… transected plantaris tendon. **b** (AGMA)… transected anterior gastrocnemius muscle aponeurosis; (PT)… transected plantaris tendon; (GIAR)… gastrocnemius intramuscular aponeurosis recession; (CF)… crural fascia; (SN)… preserved sural nerve; (GSV)… great saphenous vein.

**Fig. 5:**
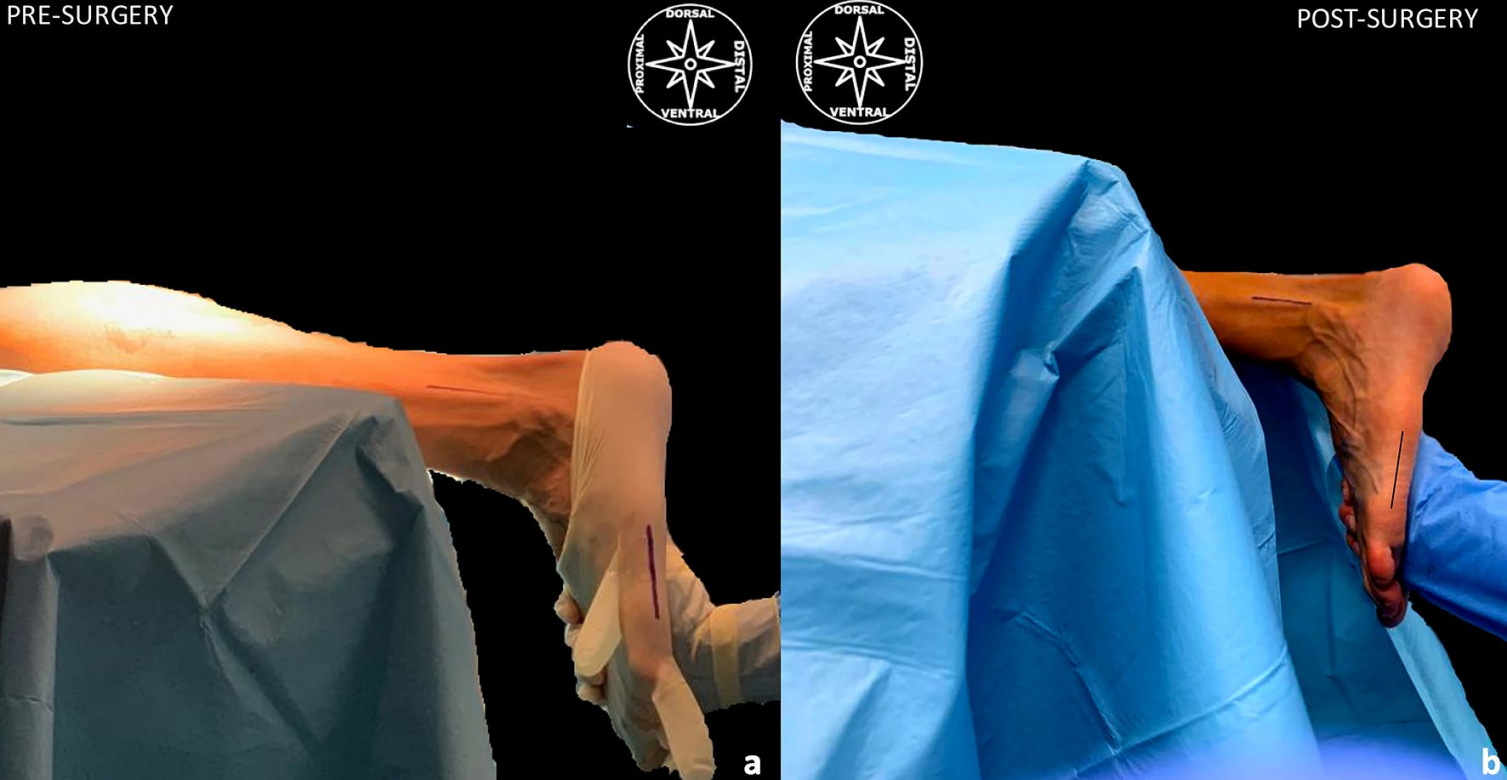
**a**, **b** The gain at the ankle joint range-of-motion (ROM) after our US-guided GIAR-technique. (black lines)…the line drawn on the distal crural region marks the shaft of the fibular bone, the black line drawn on the foot marks the shaft of the fifth metatarsal bone; a pre-surgery, clinical evaluation for equinus deformity due to gastrocemius muscle contracture. **b** post-surgery, one can see the gain in extension range of motion at the ankle joint (color figure online)

For this study, an ultrasound-guided surgical approach on ten fresh-frozen specimens (10 donors, 8 male, 2 females, 5 left and five right) was performed. The individuals had given their written informed consent prior to death for their use for scientific and educational purposes and donated their bodies to the University Complutense of Madrid (Center of Body Donation). According to National Law, scientific institutions (in general Institutes, Departments or Divisions of Medical Universities) are entitled to receive the body after death mainly by means of a specific legacy, which is a special form of last will and testament. No bequests are accepted without the donor having registered their legacy and been given appropriate information upon which to make a decision based upon written informed consent (policy of ethics) [16, 20]; therefore, an ethics committee approval was waived [16, 20].

Our inclusion criteria were: all donated bodies to the science of both sexes. 10 feet, 8 male, 2 females, 5 left and 5 right have been included with a selective gastrocnemius muscle tightness. The exclusion criteria of the donated bodies to science were: BMI above 35 (impaired ultrasound echogenicity), signs of traumas in the ankle and crural region, a history of ankle or foot ischemic vascular disorder, surgery or space-occupying mass lesions and signs osteoarthritis or bony prominences at the anterior-dorsal ankle joint.

The equipment used performing the minimally invasive ultrasound-guided gastrocnemius-intramuscular aponeurotic recession were as follows (Fig. 1): high-frequency ultrasound with 17Mhz linear probe (Sonoscape, Italy, p-50), 18-gauge needle and a syringe of 50 cc, V-shape gouges of 1 and 2 mm and 5 cm length, a 3 mm hooked knife (Acufex®), a caliper, an analogic goniometer, a pen and a buttoned probe.

The surgical procedures were performed by two podiatric surgeons with more than 6 years of experience in ultrasound-guided procedures under the guidance of a clinical anatomist, trained in clinical anatomy and also surgery for more than 10 years.

Descriptive statistic has been used to evaluate all quantitative variables.

## Methods (Figs. 1, 2, 3, 4, 5)

The specimens were positioned in a supine decubitus position and every specimen´s ROM at the ankle joint has been evaluated with the knee extended using an analogic goniometer. The proximal arm of the goniometer followed the distal third of the fibular shaft, while the distal arm followed the long axis of the fifth metatarsal shaft. The measures obtained applying a slight dorsiflexion force (about 2–5 kg) while inverting the forefoot to look the midtarsal joint were attempted three times and the mean value has been recorded [17].

The sonoanatomy of this region had been evaluated in detail before surgery in all ten specimens, the anterior gastrocnemius muscle aponeurosis thickness was measured three times using US device calipers and the mean results were recorded. The gap obtained after the anterior gastrocnemius muscle aponeurosis release and its width were measured using a flexible centimeter directly after post-surgical dissection. Measurements for all quantitative variables were obtained by the same researcher.

### Technical procedure

Using a surgical pen, under ultrasound guidance, the course of the great saphenous vein, its posterior communicating vein and nerve, the gastro-soleus medial interval, the medial and lateral gastrocnemius muscle heads, its myotendinous junction, the course of the plantaris tendon (if present) and the saphenous nerve, to get a topographical representation of the main anatomical structures, were drawn on the skin. The surgical portal point was drawn just above the myotendinous junction, as described by Bauman, and more in detail proximal to the distal “transection zone point” described by Blitz et al. [8], to optimize the biomechanical advantages, at the gastro-soleus medial interval. Depending on the lateral head of the gastrocnemius, if its myotendinous junction originates proximally to the medial one, it seems logical to draw the surgical line slightly oblique, distal-medial, as it has been described previously [8]. (Fig. 2).

### Hydrodissection step

The next step consisted in introducing a 18-gauge atraumatic needle at the "surgical portal” previously drawn right at the interval between the anterior gastrocnemius muscle aponeurosis and the posterior soleus muscle aponeurosis; as this interval has been described as a "virtually closed space" we performed—using the 18-g needle—the US-guided hydrodissection making sure that the tip of the needle stayed ventrally to the anterior gastrocnemius muscle aponeurosis. The needle enters the medial edge just laterally to the crural fascia at the medial aspect of the leg and ventrally to the medial head of the GM, proximally to the conjoint tendon, thus reaching the most lateral aspect of the anterior gastrocnemius muscle aponeurosis, ventral to the lateral head of the GM, medially to the crural fascia at the lateral aspect of the leg, crossing the intermuscular gastrocnemius septum. The hydrodissection leads to a separation of the layers and increases the “working space” at the entire interval between those two aponeuroses (if the patient has a wide aponeurosis, the assistant could introduce another syringe at the lateral anterior gastrocnemius muscle aponeurosis to hydrodissect the most lateral aspect of the aponeurosis). After hydrodissecting, the needle was left under the surgical line as an ultrasound “guide”.

### Surgical step

Using the needle as a guide, under continued US-guidance, we introduced the V-shape gouge in increasing order of size from the same portal to enlarge the “working space” to insert the hook knife.

Thus, after creating a safe channel that allowed us to introduce a 3 mm hooked knife using a 2 mm V-shape gouge as a guide, ventrally to the anterior gastrocnemius muscle aponeurosis, we kept particular attention to maintain the hook parallel to the anterior gastrocnemius muscle aponeurosis and with the cut looking proximally, then, once the fascia cruris has been perforated, turning it perpendicular with the “cutting edge” towards the anterior gastrocnemius muscle aponeurosis. Moreover, we pulled back the 2 mm V-shape, carefully introducing the retrograde knife up to the most lateral aspect of the anterior gastrocnemius muscle aponeurosis, thus, with the assistant maintaining the foot and ankle in extension and under US-guidance, then performed a retrograde cut of the anterior gastrocnemius muscle aponeurosis pushing the retrograde knife “towards” the skin to cut the thick aponeurosis and at the same time trying to do not transect red fibers.

Before pulling back the retrograde knife we easily localized the plantaris tendon following our previous drawn landmarks, therefore, under the US- guidance we hooked it and performed its transection. Finally, after extracting the hook knife we introduced the buttoned probe all the way through the surgical line under short-axis US-guidance, pushing it towards the skin to ascertain that the anterior gastrocnemius muscle aponeurosis was cut entirely. With the buttoned probe we took particular attention to check if the intermuscular septum between the medial and lateral heads of the gastrocnemius muscle and the most medial and lateral aspects of the anterior gastrocnemius muscle aponeurosis has been transected as well. If some structures or parts of them were not successfully transected the transection was repeated again (Fig. 3).

The buttoned probe has been left inside all the way through the surgical line to get the proof of our instruments course.

### Postsurgical anatomical findings (Figs. 4a, b)

After the surgical procedure was completed, all specimens have been dissected by the clinical anatomist. The dissection started from the medial crural region to the lateral aspect to ascertain the effectiveness and safety of the technique. The skin and the subcutaneous fat have been cutted off, the crural fascia has been incised, and the intermuscular space between the gastrocnemius and the soleus has been elevated to expose the anterior gastrocnemius muscle aponeurosis and the plantaris tendon for verification of a complete release. Moreover, the saphenous nerve and its accompanying great saphenous vein medially were intended to be preserved, as well as the sural medial and lateral communicating branches dorsally. We could, therefore, exclude every possible iatrogenic damage.

## Results

After a cautious dissection, a complete cut of both the anterior gastrocnemius muscle aponeurosis and the plantaris tendon could be verified. No iatrogenic damages at the great saphenous vein, saphenous nerve nor both medial and lateral sural communicating branches has been detected. So, no damages of important anatomical structures could be found.

Therefore, it has been also ascertained that the anterior gastrocnemius muscle aponeurosis was entirely transected in 10 over 10 specimens, with a mean portal length of 2 mm (± 1 mm). (Figs. 4a, b).

The average duration of the procedure, including surgical steps, within our cluster of ten specimens was approximately 16 min (± 5 min), decreasing in time for each procedure due to the learning curve: from 30 min for the first procedure to 13 min for the last one.

The mean gain at the ankle joint ROM after the GIAR was 7.9° (± 1.1°). (Fig. 5).

Little amount of red fibers of the gastrocnemius muscle, closely adjacent to the anterior gastrocnemius muscle aponeurosis were transected as well. The mean gap measured via US guidance obtained after the GIAR at the anterior gastrocnemius muscle aponeurosis was 12 mm (± 5 mm). The mean dorso-ventral thickness of the anterior gastrocnemius muscle aponeurosis was about 1.3 mm (± 0.3 mm) and its medio-lateral width was about 109 mm.

(± 11 mm). (Table 1).

**Table 1:**
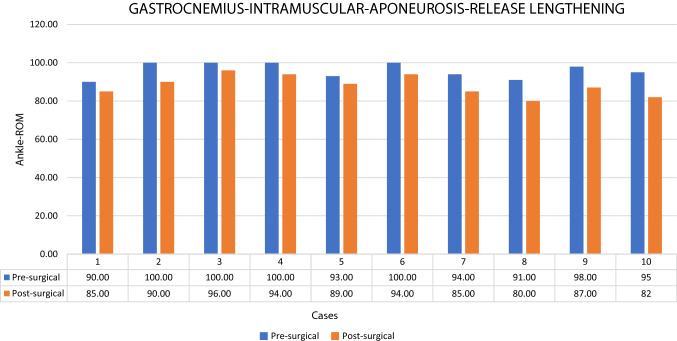
The table shows the ankle range-of-motion (Ankle-ROM) of all cases pre- and post-surgery

## Discussion

The main objective of this study was to assess the anatomical landmarks for a safe sonoguided mini-invasive procedure and to prove the effectiveness and safety of a minimally-invasive, ultrasound-guided (Baumann) surgery for the lengthening of the anterior gastrocnemius muscle aponeurosis, also re-named by Blitz et al. by the acronym “GIAR”: Gastrocnemious -intramuscular-aponeurosis-recession [7].

It has been already seen that endoscopic minimally invasive procedures for a gastro-soleus complex (GSC) release are feasible either at GSC lengthening level 3 (Strayer) and 4 (Baumann) and leads to lesser rates of complications [4].

Already in 2009 Vorha et al. have been described as an ultrasound-guided surgery for a podiatric condition performing an ultrasound-guided plantar fascia release [32]. Therefore, they should be considered the first pioneers who described an ultrasound-guided surgery that has ever been published for any foot and ankle disorder [32]. The first ultrasound-guided gastrocnemius release itself has been described in 2016 by Villanueva et al. They described a Strayer procedure in 22 specimens and in 23 patients complaining of an isolate gastrocnemius contracture, evaluating a 14 degrees mean ankle ROM gain, with no sural nerve or vessel damages through a portal of less than 2 mm only [15]. This US-guided Strayer procedure appeared to be safer compared to the endoscopic Strayer-technique due to possible sural nerve transection [29]. This level 3 procedure targets the gastrocnemius tendon distal to its myotendinous junction [5, 14]. Other anatomical studies published in 2007 and 2008 by Blitz et al. showed the high variability of the location of the fusion of the two aponeuroses (conjoint junction) [7, 8], leading the Strayer technique to a possible aspecific aponeurosis release of both the gastrocnemius and of the soleus aponeurosis, theoretically producing the risk of a complete detachment of the gastrocnemius with the risk of a complete consequent muscular atrophy and loss of function [7, 8]. Moreover, during the Strayer procedure all surgical instruments are far between the sural nerve and his accompanying lesser saphenous vein, existing in tremendous anatomical variations [23, 28], enhancing the risk of neurovascular injuries [31].

On the other hand, the GIAR seems to have more constant anatomical landmarks as it is performed proximally to the myotendinous junction of the gastrocnemius muscle. It permits to fix the pathological contractures, lengthening the aponeurosis, maintaining some degree of force during foot plantarflexion, at the same time avoiding muscle atrophy and weakness at the gastrocnemius muscle, which seems unavoidably in the complete Strayer procedure, either open, endoscopic or US-guided, respectively [15, 19, 24, 26].

Moreover, as the GIAR procedure is covered dorsally by the bellies of the gastrocnemius muscle and the crural fascia itself, the risk of damaging the sural nerve and accompanying vessels might be smaller than during the endoscopic procedure [19].

Nevertheless, either for the open and the endoscopic GIAR procedure there have been described some saphenous nerve and/or great saphenous vein damages despite their constant anatomy, leading to complications [28]. Performing the endoscopic GIAR in obese patients showed that in some cases the interval between the gastrocnemius and soleus muscles is difficult to identify, leading the surgeon to widen the portal to ensure the correct surgical spot, losing the “minimally invasive” intention [19].

Performing our novel US-guided GIAR the entry portal at the interval between the gastrocnemius and soleus muscle, the preservation of the saphenous nerve and the greater saphenous vein are ensured due to the direct visualization by ultrasound. Using the same surgical materials as for the ultrasound-guided tarsal tunnel release, previously described by our group [13, 21, 22], the small surgical portal (2 mm mean), might lead to better cosmetical results if compared to the endoscopic GIAR [19]. Ultrasound-guided surgery has a major advantage with respect to open or endoscopic procedures because it might be performed without hemostasis, less post-operative pain and leads to less post-operatives complications [13, 21]. Therefore, it also could be a possible approach for anticoagulated patients.

In their anatomical work concerning the “ideal” transection zone for the GIAR Blitz et al. claimed that one should release the anterior gastrocnemius aponeurosis proximally with regard to the confluence of the medial and lateral muscle heads (distal point of the intermuscular septum) on the dorsal surface of the gastrocnemius anterior aponeurosis [8].

This transection zone could be variable depending on the shape and the height at which the gastrocnemius heads insert [8]. In some cases, it is difficult to clinically determine the distal.

"transection zone " point, putting the lateral sural cutaneous nerve under risk of damage, because the lateral gastrocnemius muscle head is not protecting it dorsally, due to a proximal myotendinous junction. Therefore, our ultrasound-guided GIAR technique seems more stable, less invasive, precise and safe.

Another objective of our study was to measure the lengthening produced through the transection of the anterior gastrocnemius aponeurosis. Our results showed a mean gap of 12 mm (± 5 mm). These are similar to other donated bodies to science studies [1, 7, 25]**.**

Key point in the effectiveness of the GIAR procedure is the selective cut of the collagen fibers of the aponeurosis, including the transection of the intermuscular gastrocnemius septum and the plantaris tendon, sparing the red fibers [12].

In our opinion, the most challenging aspect and a possible limit of our novel technique is the reproducibility: one needs major skills and experience in ultrasonography, it seems having a long learning curve and, therefore, training is imperative.

## Conclusion

This (sono-)anatomical study of our mini-invasive, ultrasound-guided, anterior gastrocnemius muscle aponeurosis release (GIAR) shows that a complete release of the anterior gastrocnemius muscle aponeurosis was possible with a mean portal length of 2 mm only and a mean gain at the ankle joint ROM of 7.9° without damaging important anatomical structures. These results indicate that our novel ultrasound-guided (Baumann) surgery for the lengthening of the anterior gastrocnemius muscle aponeurosis might be an effective and safe procedure.
